# Identifying Actionable Variants Using Capture-Based Targeted Sequencing in 563 Patients With Non-Small Cell Lung Carcinoma

**DOI:** 10.3389/fonc.2021.812433

**Published:** 2022-02-04

**Authors:** Haiping Jiang, Yinan Wang, Hanlin Xu, Wei Lei, Xiaoyun Yu, Haiying Tian, Cong Meng, Xueying Wang, Zicheng Zhao, Xiangfeng Jin

**Affiliations:** ^1^ Department of Oncology, The Affiliated Hospital of Qingdao University, Qingdao, China; ^2^ Department of Obstetrics and Gynecology, Peking University Shenzhen Hospital, Shenzhen, China; ^3^ Department of Thoracic Surgery, The Affiliated Hospital of Qingdao University, Qingdao, China; ^4^ Research and Development Department, Shenzhen Byoryn Technology Co., Ltd, Shenzhen, China

**Keywords:** targeted therapy, EGFR, capture-based targeted sequencing, NSCLC, mutation

## Abstract

Although the NSCLC diagnostic standards recommend the detection of driver gene mutation, comprehensive genomic profiling has not been used widely in clinical practice. As to the different mutation spectrum characteristics between populations, the research based on Chinese NSCLC cohort is very important for clinical practice. Therefore, we collected 563 surgical specimens from patients with non-small cell lung carcinoma and applied capture-based sequencing using eight-gene panel. We identified 556 variants, with 416 potentially actionable variants in 54.88% (309/563) patients. These single nucleotide variants, insertions and deletions were most commonly found in *EGFR* (55%), followed by *ERBB2* (12%), *KRAS* (11%), *PIK3CA* (9%), *MET* (8%), *BRAF* (7%), *DDR2* (2%), *NRAS* (0.3%). By using ten protein function prediction algorithms, we also identified 30 novel potentially pathogenic variants. Ninety-eight patients harbored EFGR exon 21 p.L858R mutation and the catalytic domain of the protein tyrosine kinase (PTKc) in *EGFR* is largely mutated. In addition, there were nine frequent pathogenic variants found in five or more patients. This data provides the potential molecular basis for directing the treatment of lung cancer.

## Introduction

Non-small cell lung carcinoma (NSCLC) is the leading factor of cancer death rate worldwide (18.4% of the total cancer deaths) (1). With the emergence of targeted therapies for NSCLC, genetic testing has become a mandatory component for guiding the patient treatment (2). NSCLC diagnostic standards has included the detection of epidermal growth factor receptor (*EGFR*), B-Raf proto-oncogene (*BRAF*), MET proto-oncogene (*MET*), erb-b2 receptor tyrosine kinase 2 (*ERBB2*), and erb-b2 receptor tyrosine kinase 2 (*KRAS*) mutations (3). However, comprehensive genomic profiling has not been used widely in clinical practice.

For the time being, laboratory work about therapeutic targeted genes of NSCLC has been undertaken in many countries. A study sequenced whole-exome genome in 31 NSCLCs and identified common and unique mutation spectra (4). Hu et al. performed a genome-wide association scan in 2,331 lung cancer patients and found four related SNPs (5).Si et al. described low‐frequency gene alterations by next-generation sequencing (NGS) (6). NGS sequencing maybe more sensitive to detect actionable genomic alterations (7, 8).

Despite previous studies have identified and discovered many lung cancer driver genes, the mutation spectrum characteristics of NSCLC patients is different between populations. The research of genomic characteristics of a large-scale Chinese NSCLC cohort is very important for clinical practice. Therefore, we conducted a retrospective study to use a hybrid capture-based eight-gene panel NGS assays to detect driver genes in tumor surgical specimens from patients with NSCLC. Among the eight target genes, *EGFR*, *BRAF*, *MET*, *ERBB2*, and *KRAS* are lung cancer target therapy-associated genes recommended by NCCN Clinical Practice Guidelines in Oncology. Neuroblastoma RAS viral (v-ras) oncogene homolog (*NRAS*), phosphatidylinositol-4,5-bisphosphate 3-kinase, catalytic subunit alpha (*PIK3CA*), and discoidin domain receptor tyrosine kinase 2 (*DDR2*) are supported by clinical trials, literature, and *in vitro* evidence. We intended to confirm the clinical feasibility and utility of the capture-based targeted sequencing in reflecting the genetic profiles and assisting clinicians in clinical decision-making. Moreover, 30 novel potentially pathogenic variants we identified emerged as a potential therapeutic target for NSCLC.

## Materials and Methods

### Samples Collection

We collected 563 tissue biopsy samples from 563 patients with NSCLC. Clinical diagnosis was verified by cytopathology. The Ethics Committee of the Affiliated Hospital of Qingdao University approved the study (approval no.QYFY WZLL 26620). All patients provided signed informed consent. We performed the experiments according to the guideline released by the National Health and Family Planning Commission of the PRC.

### Biopsy DNA Extraction

According to the manufacturer’s instructions, DNA was extracted from tissue biopsy samples using the QIAamp DNA formalin-fixed paraffin-embedded (FFPE) tissue kit (Qiagen). Cell-free DNA was isolated from plasma using the QIAamp Circulating Nucleic Acid kit (Qiagen). DNA integrity, purity, and concentration were assessed by agarose gel electrophoresis, the NanoDrop2000 spectrophotometer, and the Qubit 2.0 fluorimeter (Thermo Fisher Scientific). Qualified DNA samples were used for library construction.

### Library Construction and Sequencing

Library construction was performed as previously described (9). DNA was fragmented randomly using ultrasound, followed by end repair, phosphorylation, and adaptor ligation. Fragments were hybridized with a custom sequence capture-probe (Nimblegen, USA), amplified through PCR, and sequenced on an Illumina Hiseq2500 platform with 2×101 bp paired-end reads (Illumina, San Diego, USA). The panel enables capture-based ultra-deep targeted sequencing for the following genes: *EGFR*, *KRAS*, *MET*, *BRAF*, *NRAS*, *PIK3CA*, *DDR2*, and *ERBB2* (**Supplementary Table 1**).

### Sequencing Data Analysis

**Figure 1:**
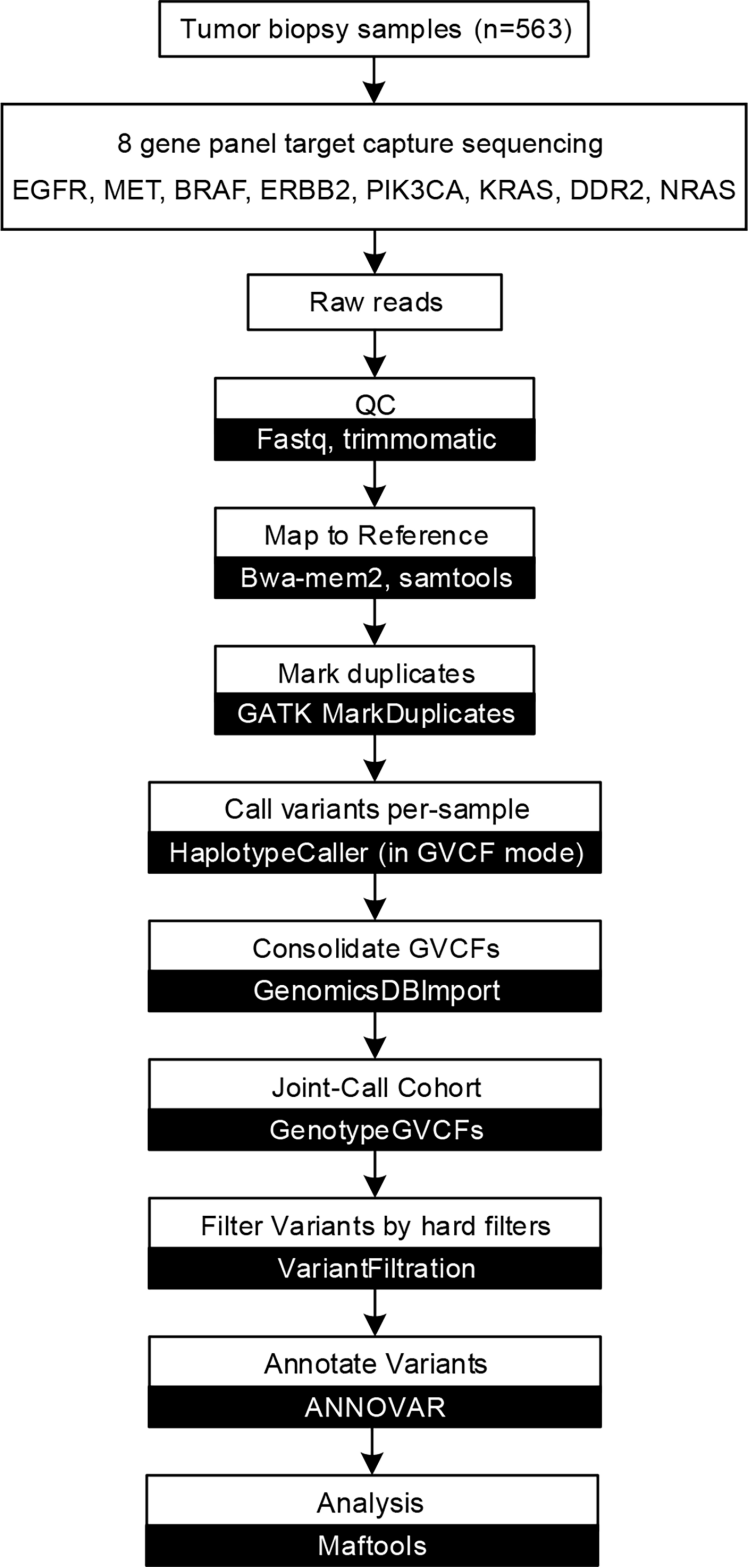
Capture-based targeted sequencing data analysis flow chart. Data analysis flow chart of described methods and analyses. White boxes represent data processes, while black boxes represent software.

After obtaining raw sequencing data, SOAPnuke (http://soap.genomics.org.cn/) (10) were used to remove adaptors and filter low-quality reads. Clean reads were mapped to the human genome (hg38) by using bwa-mem2 (https://github.com/bwa-mem2/bwa-mem2) (11). GATK (v 4.1.9.0) (12) was used to remove PCR duplication, call variants, and filter variants with the following hard-filtering expressions for single nucleotide variants (SNVs): “QD < 2.0”, “MQ < 40.0”, “FS > 60.0”, “SOR > 3.0”, “MQRankSum < -12.5”, and “ReadPosRankSum < -8.0”. As for insertions and deletions (indels), the filtering conditions were “QD < 2.0”, “ReadPosRankSum < -20.0”, “InbreedingCoeff < -0.8”, “FS > 200.0”, and “SOR > 10.0”. Then, loci with a depth less than 50 were filtered out by VCFtools (v4.2) (13). Loci not detected in at least 50% of samples were filtered and discarded. ANNOVAR (http://www.openbioinformatics.org/annovar/) (14) was used to annotation the remaining variants based on population databases to exclude polymorphisms, cancer-specific variant databases to interpret clinical significance, and algorithms to predict the functional impact of sequence variant/splice site changes. We filtered SNVs by the following rules: 1) variants with population frequencies higher than 1% were classified as single nucleotide polymorphisms (SNPs) and excluded from further analysis according to the Exome Aggregation Consortium dataset (ExAC, http://exac.broadinstitute.org), 1000 Genomes Project (http://www.1000genomes.org/) (15), and ESP6500SI-V2 database; 2) variants beside exonic or splicing region were filtered out. We subsequently predicted the pathogenicity of the SNVs by SIFT (16), Polyphen2 (HDIV/HVAR) (17), LRT (18), MutationTaster (19), MutationAssessor (20), FATHMM (21), FATHMM-MKL, PROVEAN (22), MetaSVM/LR (23) and M-CAP (24). Domain definitions were used from the InterPro domain database (release 86.0, http://www.ebi.ac.uk/interpro). ClinVar (http://www.ncbi.nlm.nih.gov/clinvar) (25), Catalog of Somatic Mutations in Cancer (COSMIC, http://cancer.sanger.ac.uk/cosmic) (26), and dbSNP (http://www.ncbi.nlm.nih.gov/projects/SNP/) (27) were used to evaluate clinical significance of variants. We also employ OncoKB (http://oncokb.org/) (28) and OncoVar (https://oncovar.org/) (29) to identify actionable mutations and driver mutations, respectively. The workflow is shown in **Figure 1**. We used R (v4.0.5) package maftools (https://github.com/PoisonAlien/maftools) (30) to visualize mutations and analyze the mutual exclusivity of variants.

### Statistical Analysis

We presented frequency and percentage for descriptive statistics. We used pair-wise Fisher’s exact test, which was performed with GraphPad Priem 8 (https://www.graphpad.com), to compare the difference between the rates of affected cases in The Cancer Genome Atlas (TCGA) cohort and this cohort. We also used maftools to perform Fisher’s exact test to detected mutually exclusive or co-occurring set of genes. P < 0.05 was considered statistically significant.

## Results

### Sequencing and Variant Detection

**Figure 2:**
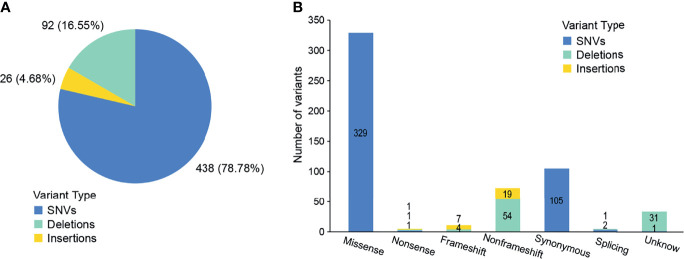
Outcomes of variants calling in NSCLC patients. **(A)** Percentage of SNVs and indels identified by next-generation sequencing. **(B)** The number of variants in each category.

We conducted a retrospective nationwide study to survey the prevalence rate of driver gene mutations in advanced (stage IIIB to IV) Chinese NSCLC patients with various histological subtypes. We conducted capture-based targeted sequencing of 563 primary lung tumors samples. On average, we generated 8.4 Gb of sequence per sample to a mean average depth of ~1,810×. To reduce false-positive rates and eliminate common germline mutations, we adopted comprehensive filtering criteria and removed variants with population frequencies higher than 1% in dbSNP, ExAC, 1000 Genomes, and ESP6500SI-V2 database. After filtering, we identified 556 variants in 354 (62.88%) samples (**Supplementary Table 2**), including 438 SNVs, 26 insertions, and 92 deletions (**Figure 2A**). Among them, 416 variants were loss of function (LOF) in 309 samples, i.e., 329 missense SNVs, three nonsense SNVs, 84 indels, and three splicing variants (**Figure 2B**). Missense SNVs and frameshift indels generally lead to the inactivation of the protein products. So these variants may be clinically significant and were included in the subsequent analyses.

### Mutation Spectrum of 354 NSCLC Patients

**Figure 3:**
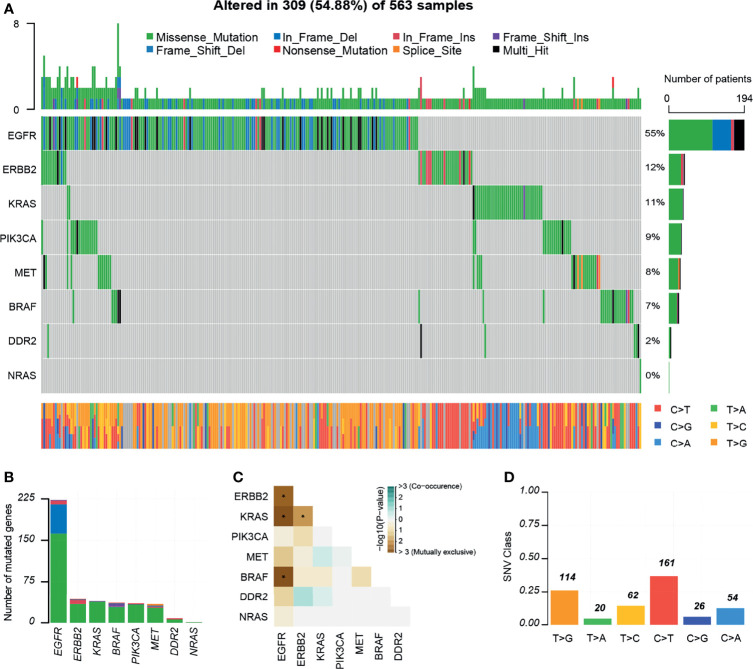
Mutations landscapes in NSCLC patients. **(A)** Significantly mutated genes of 309 patients with NSCLC. Top, the number of mutations of each sample. Middle, targeted genes are ranked based on the mutation frequency. Different colors represent different types of mutation. Variants annotated as Multi_Hit are those genes which are mutated more than once in the same sample. Right, the percentages of genes with mutations. Bottom, the class of SNVs for each sample. **(B)** Number of mutated genes. **(C)** Mutually exclusive or co-occurring set of genes. **(D)** SNV class. INS, insertions; DEL, Deletions. *P < 0.05.

In total, 309 (54.88%) of the patients had at least one variant, 297 of which had one or more potentially actionable variants (**Figure 3A**). According to the OncoVar database, 193 variants were assessed as driver variants for lung adenocarcinoma and 25 variants for lung squamous cell carcinoma. The most common driver mutations were in *EGFR* (55%) followed by *ERBB2* (12%), *KRAS* (11%), *PIK3CA* (9%), *MET* (8%), *BRAF* (7%), *DDR2* (2%), *NRAS* (0.3%). We compared mutations in significantly mutated genes in NSCLC between this cohort and TCGA lung adenocarcinoma and lung squamous cell carcinoma cohort (n = 831). Notable differences from TCGA data included *KRAS* (11 *vs* 20%; P = 0.001), *EGFR* (55 *vs* 12%, P < 0.001), *DDR2* (2 *vs* 6%; P = 0.008), *ERBB2* (12 *vs* 4%; P < 0.001), and *MET* (8 *vs* 4%; P = 0.017; **Supplementary Table 3**). We detected 233 mutations in *EGFR*, 41 in *ERBB2*, 39 in *KRAS*, 34 in *BRAF* and *PIK3CA*, 29 in *MET*, eight in *DDR2*, and one in *NRAS* (**Figure 3B**). Moreover, some driver mutations demonstrated a mutually exclusive relationship, such as *EGFR*/*BRAF*, *EGFR*/*KRAS*, *KRAS*/*ERBB2* and so on (**Figure 3C**). We observed that C>T/G>A alteration was more frequent than other forms (**Figure 3D**).

### Clinical Implications of Mutations

**Figure 4:**
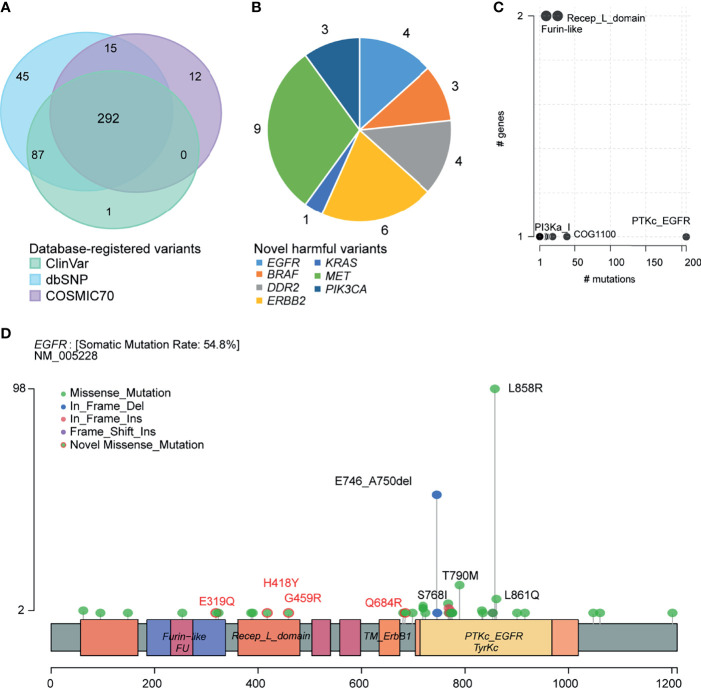
Clinical implications of mutations. **(A)** Venn plot of database-registered variants. **(B)** Novel harmful variants pie chart. **(C)** Frequently mutated pfam protein domains in NSCLC. Bubble sizes are proportional to the number of genes containing the domain. **(D)** Schematic locations of mutations in *EGFR*.

We annotated the clinical significance of these mutations based on ClinVar, dbSNP COSMIC70, and OncoKB databases. We predicted the pathogenicity using ten algorithms following the American College of Medical Genetics and Genomics and the Association for Molecular Pathology (ACMG/AMP) guidelines. In total, 380, 439, and 319 variants were annotated to the ClinVar, dbSNP, and COSMIC70 databases (**Figure 4A**). ClinVar and COSMIC70 database-registered variants (n=292) were considered functionally important mutations. After comparing with ClinVar, we classified 98 variants as pathogenic, 70 as benign, 173 as drug response, two as risk factors, and 213 as variants of uncertain significance (VUS; **Supplementary Table 4**). Among VUS, nine had conflicting evidence for pathogenic and benign criteria, while the others did not have enough evidence. In addition, 68 variants were oncogenic and 17 were likely oncogenic in the OncoKB database. Among the 104 novel variants, which were not registered in the dbSNP build 150, ClinVar, and COSMIC70, 30 SNVs were predicted as deleterious by at least three algorithms (**Supplementary Tables 2**, **5**). The gene with the highest number of novel variants was *MET* (n = 9) followed by *ERBB2* (n = 6), *DDR2* (n = 4), *EGFR* (n = 4), *PIK3CA* (n = 3), *BRAF* (n = 3), and *KRAS* (n = 1; **Figure 4B**). Among them, *ERBB2* p.R678W mutation was predicted as likely oncogenic according to the OncoKB database.

We then summarized amino acid changes to know what domain in this cohort is most frequently affected. The catalytic domain of the protein tyrosine kinase (PTKc) in *EGFR* is largely mutated (**Figure 4C**). Specifically, the *EGFR* LOF variants comprised 162 missense mutations (p.E319Q, p.H418Y, p.G459R, and p.Q684R are novel missense mutations), 53 nonframeshift deletions, seven nonframeshift insertions, and one frameshift insertion. Among them, 98 (44%, 98/223) patients harbored *EFGR* exon 21 p.L858R mutation, 53 (24%) patients harbored exon 19 p. E746_A750del, 10 (4%) patients harbored exon 18 p.G719X mutation, 13 (6%) patients harbored exon 20 p.T790M mutation, and 5 (2%) patients harbored exon 20 p.S768I mutation (**Figure 4D**). **Supplementary Figures 1–6** provided the schematic locations of mutations of the remaining seven genes. We identified four frequent pathogenic variants found in five or more patients in *ERBB2* (p.R128Q), *PIK3CA* (p.E545K), *KRAS* (p.G12V/A/C/D), and *BRAF* (p.D22N).

## Discussion

In this descriptive study, we retrospectively investigated a cohort of 563 NSCLC patients using the capture-based targeted sequencing and revealed a hotspot mutations spectrum in NSCLC. We identified 556 variants, with 416 potentially actionable variants in 54.88% (309/563) patients. The percentage of patients harboring actionable genetic alterations is slightly lower than the previous studies (62%) (31). This is due to the small target gene panel. We compared our list of mutated genes with the COSMIC, dbSNP, and ClinVar databases, then used ten protein function prediction algorithms to revealed that 30 new mutated genes found in our exome study have not yet been reported.

Otherwise, our study accurately reproduced a number of some observations in previous studies. For example, our mutation spectrum is similar to the somatic mutation spectrum of NSCLC reported in other studies (4, 32, 33). In our cohort, *EGFR* (55%) is the most common driver mutations followed by *ERBB2* (12%), *KRAS* (11%), *PIK3CA* (9%), *MET* (8%), *BRAF* (7%), *DDR2* (2%), *NRAS* (0.3%). Our data further highlight the importance of these mutations in lung tumorigenesis. The *EGFR* exon 21 p.L858R mutation is the highest-frequency mutation and the catalytic domain of the protein tyrosine kinase (PTKc) in *EGFR* is largely mutated. L858R mutation is considered sensitive to EGFR-TKIs. As expected, C>T/G>A transversions were the most common substitution in patients with lung cancer, which was the tobacco exposure-related mutation signatures (34, 35). *EGFR*/*BRAF*, *EFRF*/*KRAS*, *KRAS*/*ERBB2* demonstrated a mutually exclusive relationship, consistent with the recent evidence from cancer genomic studies which demonstrated that driver genes are often mutated in a mutually exclusive manner (36).

We compared our cohort with TCGA Caucasians cohort to explore the mutation profile in Chinese NSCLC patients, the mutation frequency of *EGFR*, *KRAS*, *DDR2*, *ERBB2*, and *MET* in our cohort is different from the frequency in TCGA cohort. This underlines the importance of the extensive analytical investigations. This study could help develop targeted treatment strategy and design gene panel which are more suitable for Chinese NSCLC patients.

There are some weaknesses in the present study that must be recognized. Firstly, because surgical specimens (n = 563) were collected over a longer period, patient clinical information was missing, which placed a somewhat large range of limitations. Secondly, we focused on point mutations and small insertions and deletions in this study. As to the complex genomic alterations of lung tumors, gene fusion and copy number variant analysis should be included in the future study. Third, we did not sequence tumors’ matched normal samples simultaneously, which resulted in the inability to distinguish between somatic and germline mutations.

In summary, we showed a clear genomic landscape of the mutation frequencies of oncogenic drivers and 30 novel potentially pathogenic variants from 563 patients with NSCLC. This data may assist clinicians in clinical decision-making and provides the potential molecular basis for directing the treatment of lung cancer.

## Data Availability Statement

The datasets presented in this study can be found in online repositories. The names of the repository/repositories and accession number(s) can be found below: https://db.cngb.org/, CNP0002486.

## Ethics Statement

The studies involving human participants were reviewed and approved by The Ethics Committee of the Affiliated Hospital of Qingdao University. The patients/participants provided their written informed consent to participate in this study.

## Author Contributions

XJ and ZZ participated in study conception and design. HX, WL,XY, HT, and CM enrolled and managed patients. HJ, HT, HX, and WL carried out collection and assembly of data. HJ, YW, and XW were involved in data analysis and interpretation. HJ and YW prepared the manuscript and manuscript figures. XJ and ZZ edited, critically read, and revised the manuscript. All authors contributed to the article and approved the submitted version.

## Conflict of Interest

Author ZZ and XW were employed by the company Shenzhen Byoryn Technology Co., Ltd.

The remaining authors declare that the research was conducted in the absence of any commercial or financial relationships that could be construed as a potential conflict of interest.

## Publisher’s Note

All claims expressed in this article are solely those of the authors and do not necessarily represent those of their affiliated organizations, or those of the publisher, the editors and the reviewers. Any product that may be evaluated in this article, or claim that may be made by its manufacturer, is not guaranteed or endorsed by the publisher.
