# Supplementary material for: Identifying Actionable Variants Using Capture-Based Targeted Sequencing in 563 Patients With Non-Small Cell Lung Carcinoma
Source: Front Oncol. 2022 Feb 4;11:812433. doi: 10.3389/fonc.2021.812433 (PMC8854177; doi:10.3389/fonc.2021.812433)

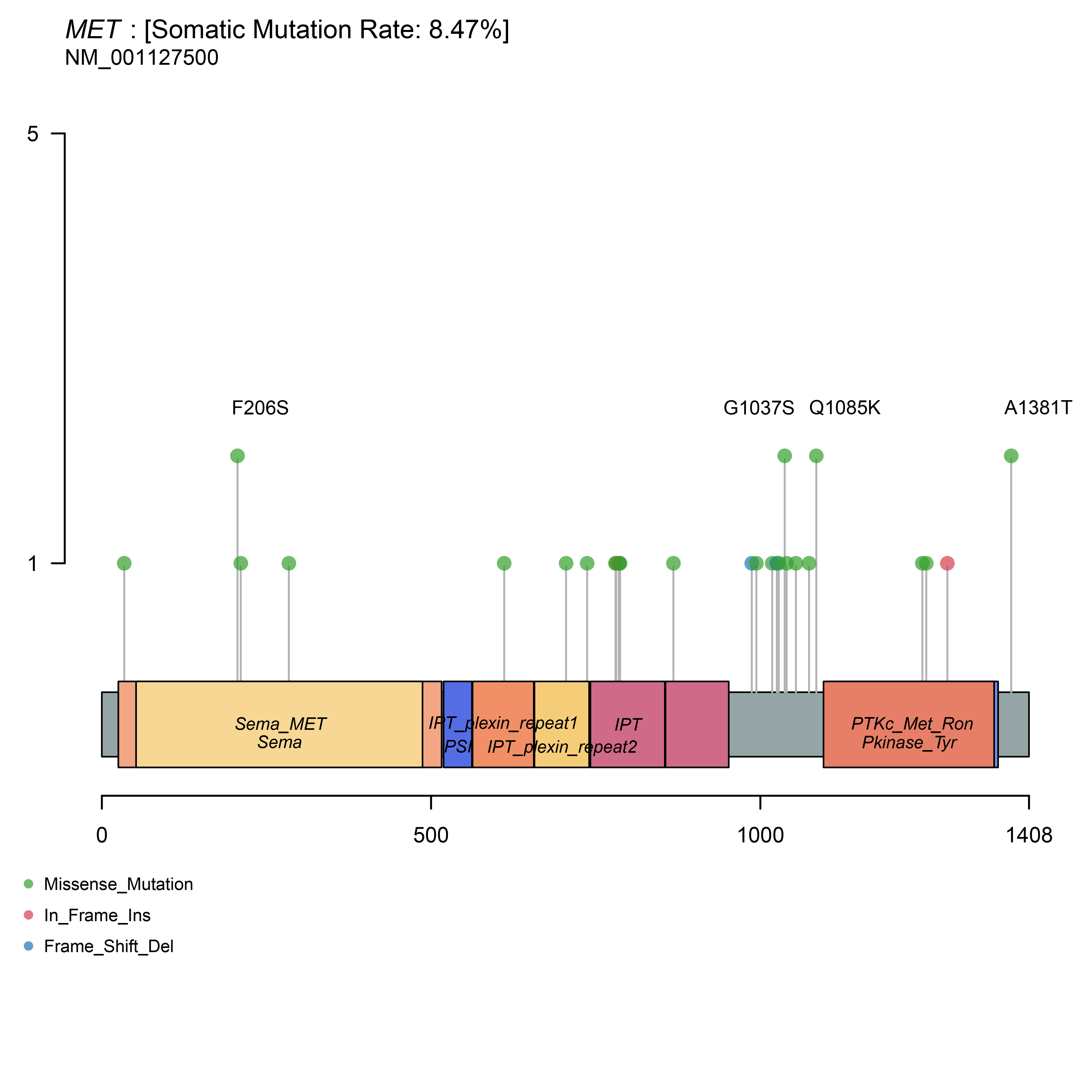


Supplementary Figure 1. Schematic locations of mutations in *MET*.


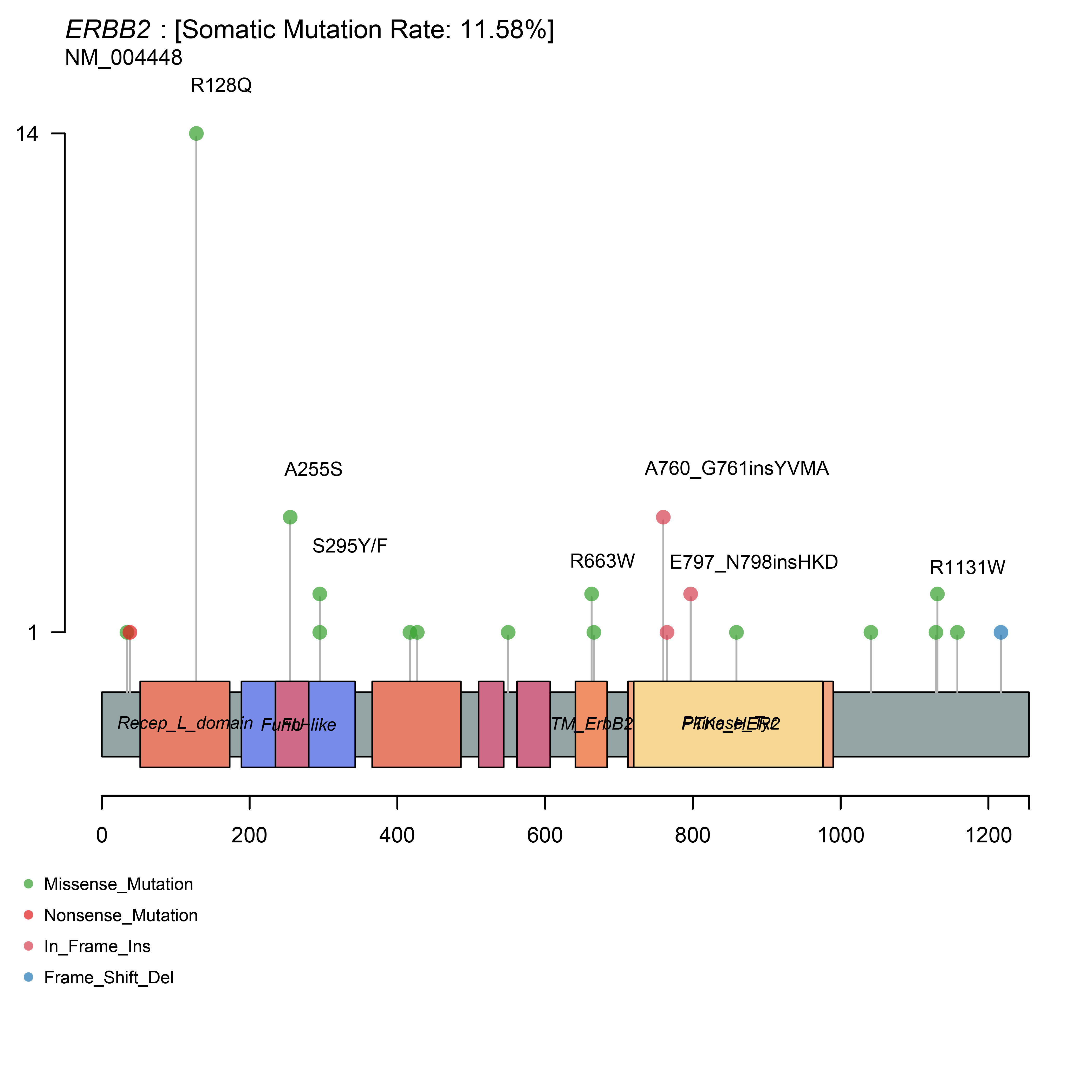


Supplementary Figure 2. Schematic locations of mutations in *ERBB2*.


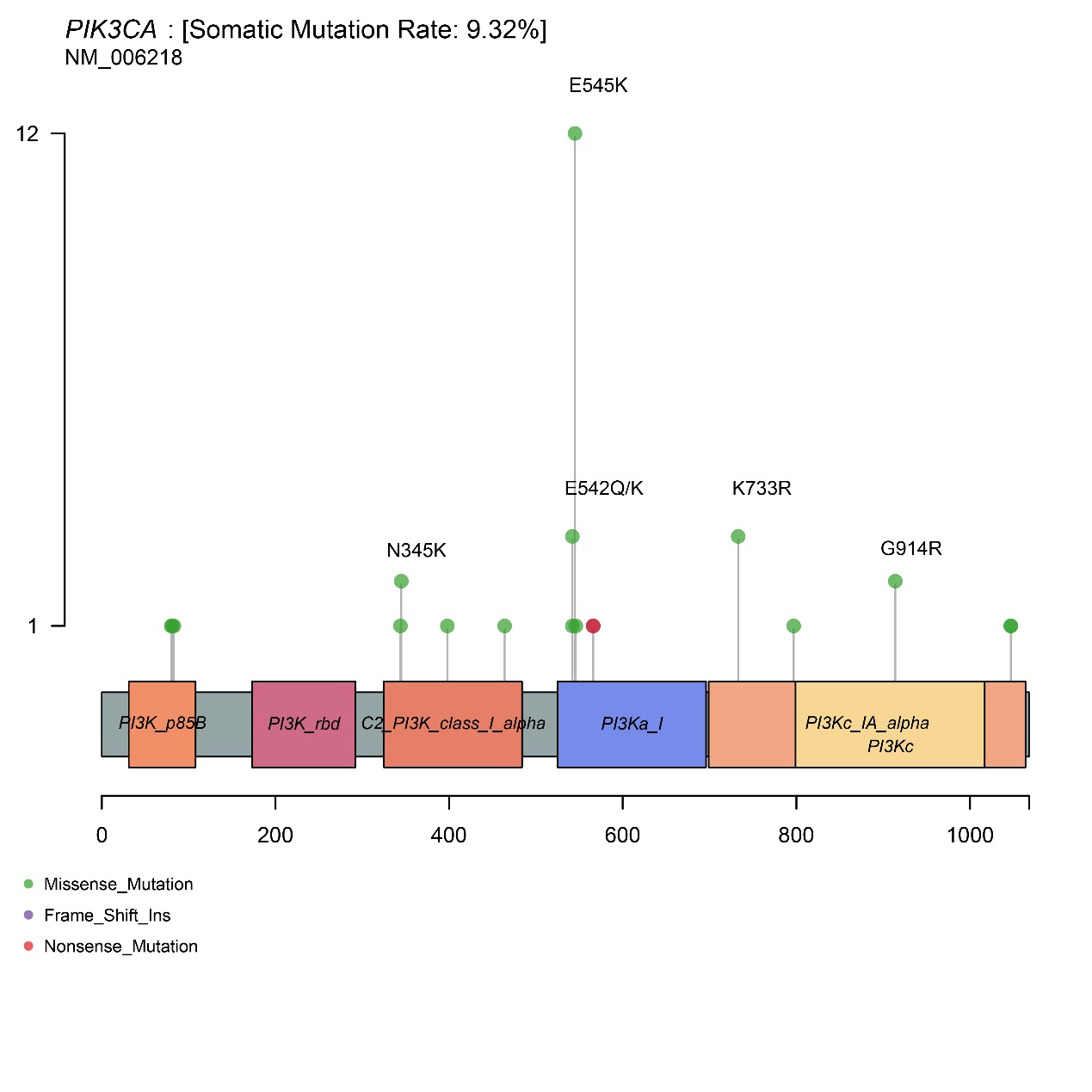


Supplementary Figure 3. Schematic locations of mutations in *PIK3CA*.


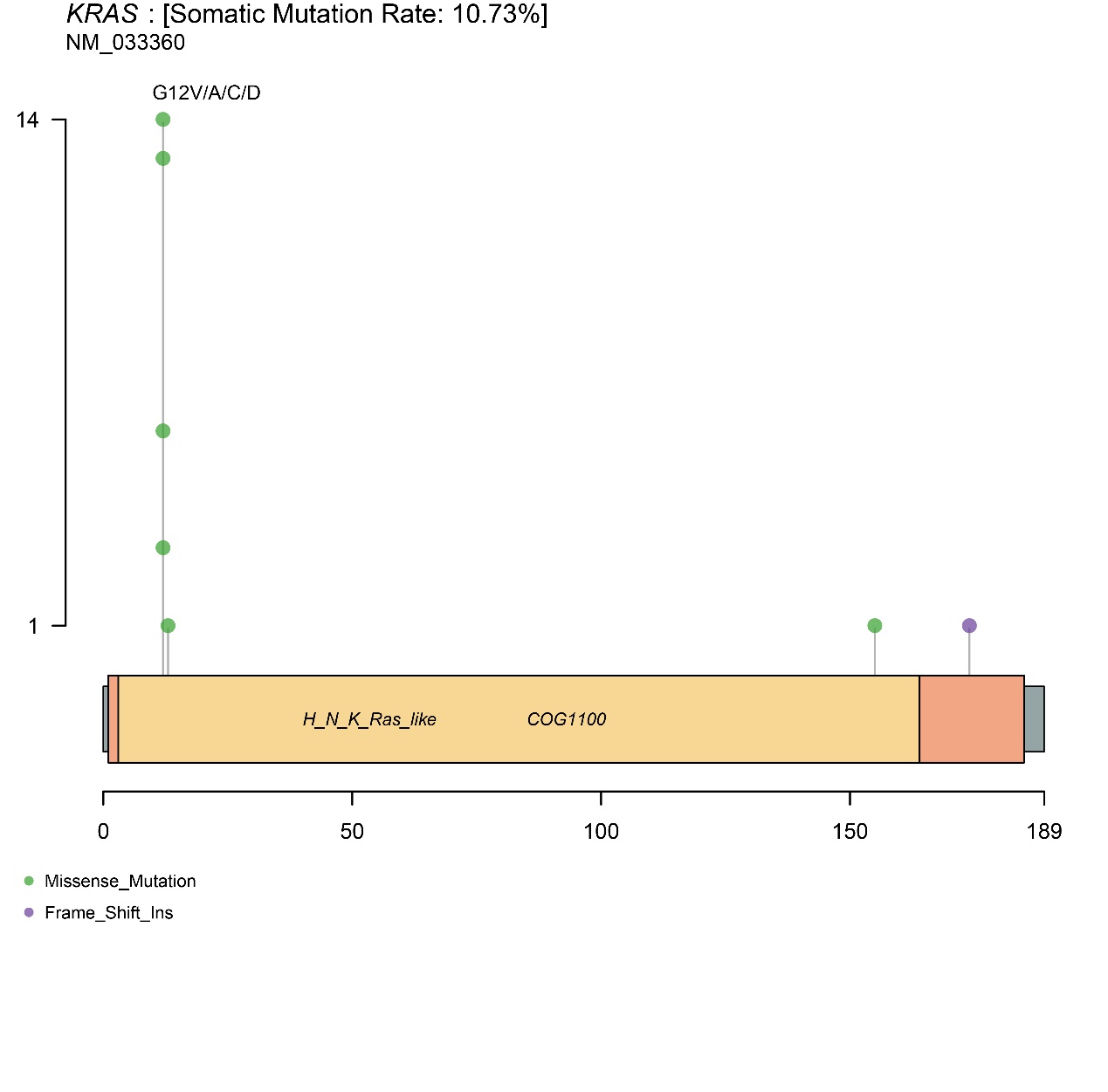


Supplementary Figure 4. Schematic locations of mutations in *KRAS*.


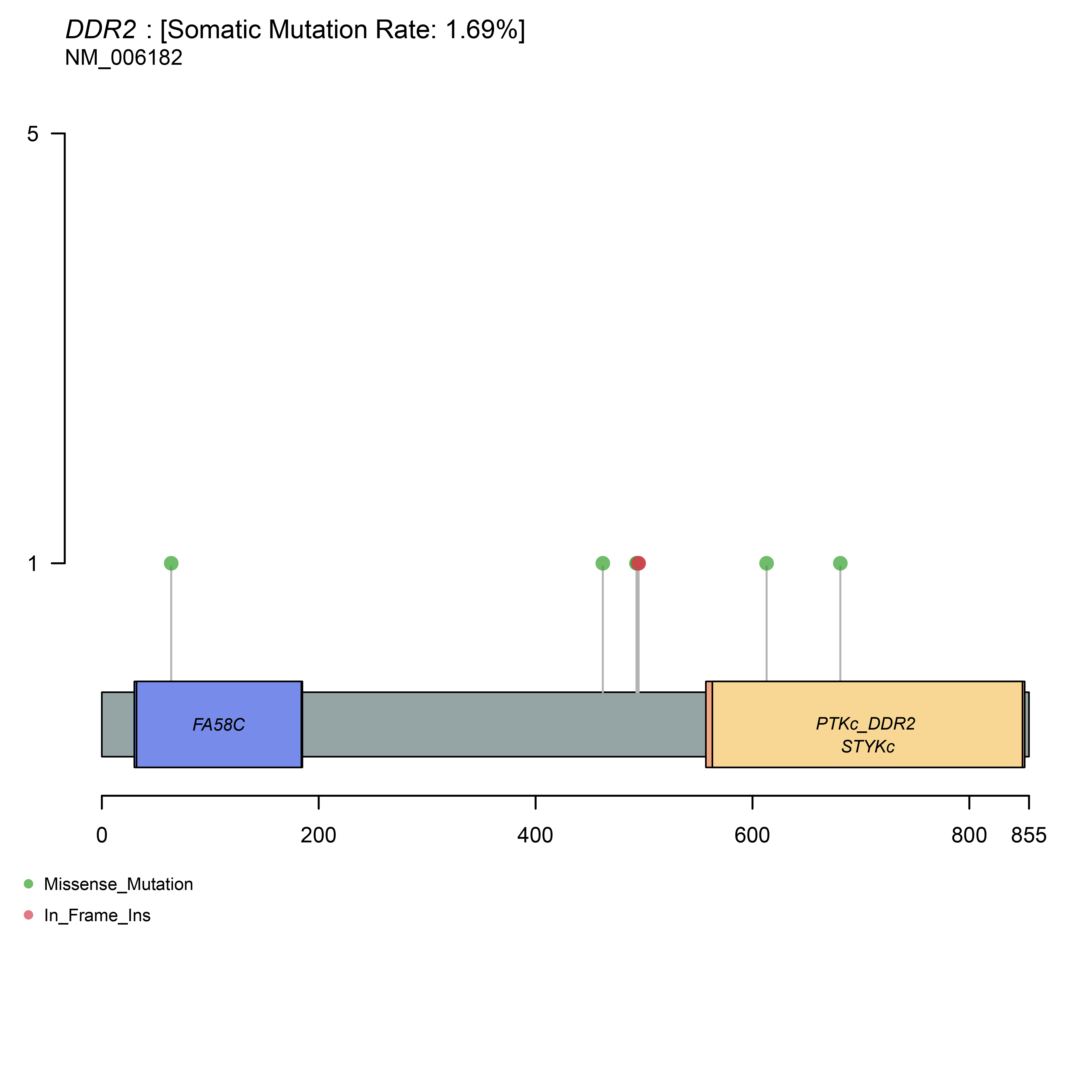


Supplementary Figure 5. Schematic locations of mutations in *DDR2*.

Supplementary Figure 6. Schematic locations of mutations in *BRAF*.


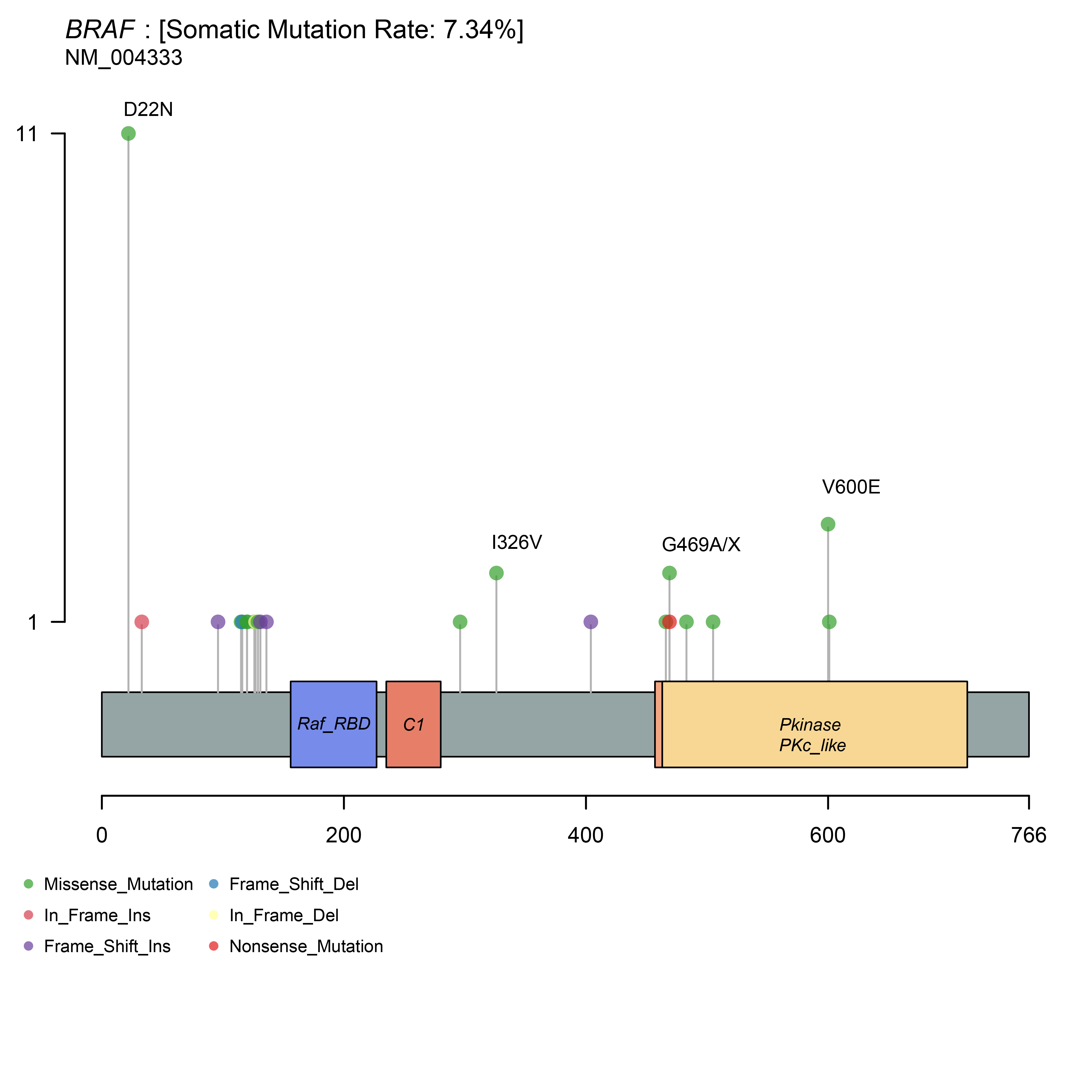

Supplement: Supplementary file 1 [file DataSheet_1.docx]
